# KinMethyl: robust methylation detection in prokaryotic SMRT sequencing via kinetic signal modeling and deep feature integration

**DOI:** 10.1093/bioadv/vbaf249

**Published:** 2025-10-09

**Authors:** Jichen Zhang, Yutaka Saito

**Affiliations:** Graduate School of Frontier Sciences, The University of Tokyo, Kashiwanoha, Kashiwa, Chiba 277-0882, Japan; Graduate School of Frontier Sciences, The University of Tokyo, Kashiwanoha, Kashiwa, Chiba 277-0882, Japan; Artificial Intelligence Research Center, National Institute of Advanced Industrial Science and Technology (AIST), Tokyo, 135-0064, Japan; School of Frontier Engineering, Kitasato University, Sagamihara, Kanagawa 252-0373, Japan

## Abstract

**Motivation:**

Accurate detection of 5-methylcytosine (5mC) from PacBio single-molecule real-time (SMRT) sequencing remains challenging in prokaryotes due to weak kinetic signals and motif diversity.

**Results:**

Here, we present KinMethyl, a generalizable deep learning framework that integrates sequence and kinetic signals to improve methylation detection across diverse bacterial genomes. Central to our approach is a regression model trained on whole-genome amplified samples to estimate the expected kinetics signals of unmethylated sequences. These predicted signals are incorporated into a downstream classifier to enhance the performance under low signal-to-noise conditions. KinMethyl outperforms existing tools such as kineticstools and ccsmeth across multiple bacterial species, methylation motifs, and modification types not only 5mC but also N6-methyladenine (6 mA) and N4-methylcytosine (4mC). In 5mC classification, KinMethyl improved the AUC by up to 0.20 compared to the existing method (0.6165 to 0.8190) with statistical significance (DeLong’s test, *P *< 1e-10). The improvements were consistently observed in cross-species evaluations as well as different sequencing platforms including RSII and Sequel. This work highlights the utility of kinetic signal modeling and feature integration for robust and motif-independent methylation analysis in prokaryotic epigenomics.

**Availability and implementation:**

The source code is available at https://github.com/ZhangBio/KinMethyl.

## 1 Introduction

DNA methylation is a key epigenetic modification involved in gene regulation and genome defense (Moore *et al.* 2013; Seong *et al.* 2021). In prokaryotes, the most common methylation types are N6-methyladenine (6 mA), N4-methylcytosine (4mC), and 5-methylcytosine (5mC), often associated with restriction–modification systems and other regulatory processes (Seong *et al.* 2021; Anton and Roberts 2021). While traditional biochemical methods such as bisulfite sequencing (Frommer *et al.* 1992), restriction enzyme assays (Hayashizaki *et al.* 1993; Khulan *et al.* 2006), and methylated DNA immunoprecipitation (Wilson *et al.* 2006) have enabled detection of DNA methylation, these indirect techniques typically require additional experimental steps, such as bisulfite conversion or methylation-sensitive restriction enzyme digestion, which increase experimental complexity. These procedures also limit scalability, as they are often restricted to specific methylation motifs and are not easily applicable to motif-diverse settings or de novo methylation detection.

The advent of third-generation single-molecule sequencing technologies, such as Oxford Nanopore Technologies (ONT) (Clarke *et al.* 2009) and PacBio single-molecule real-time (SMRT) sequencing (Eid *et al.* 2009), has enabled the direct detection of DNA modifications by capturing subtle alterations in their sequencing signals that are affected by sequence context and modified base. While Nanopore sequencing detects base modifications via shifts in ionic current (Simpson *et al.* 2017), SMRT sequencing infers DNA methylation from changes in polymerase kinetic signals, including inter-pulse duration (IPD) and pulse width (PW) (Flusberg *et al.* 2010; Feng *et al.* 2013); for example, 6 mA methylation usually causes a higher IPD in the modified base and can be detected by comparing to the IPD of an unmodified base under the same sequence context.

This study focuses on SMRT-based detection of 5mC, which remains particularly difficult to detect due to its weak kinetic signature and can be more challenging in bacterial genomes where methylation motifs are diverse. For modifications such as 6 mA and 4mC, which produce strong kinetic alterations, rule-based approaches like kineticstools (Pacific Biosciences 2013) and SMALR (Beaulaurier *et al.* 2015) have shown promising results by comparing observed signals to expected values at unmethylated sites. However, these methods are less effective for 5mC, where the kinetic differences are subtle and less distinguishable (Flusberg *et al.* 2010; Pacific Biosciences 2015). To mitigate this, methods like AgIn (Suzuki *et al.* 2016) aggregate kinetic signals from neighboring CpG sites to enhance detection sensitivity.

Recent deep learning models such as HKmodel (Tse *et al.* 2021), Primrose (Pacific Biosciences 2025), and ccsmeth (Ni *et al.* 2023) have achieved high accuracy for 5mC detection in human CpG contexts using circular consensus sequencing (CCS, also known as HiFi) reads, but these models are typically tailored to specific symmetric motifs and eukaryotic genomes (Sigurpalsdottir *et al.* 2024; Fu *et al.* 2025), making them less suitable for prokaryotic applications. Accurate detection of 5mC in bacterial genomes is particularly challenging for several reasons. First, bacterial methylation motifs are highly diverse, often species-specific, and may be non-palindromic, which makes it difficult to design motif-specific detection rules. Second, this motif diversity also leads to a wide range of sequence contexts, increasing the variability of background kinetic signals and making it harder to for machine learning models to accurately learn the baseline kinetic behavior. Third, unlike eukaryotic CpG methylation that has symmetric patterns, prokaryotic methylation is often asymmetric, reducing the signal-to-noise ratio. These factors collectively make rule-based and motif-dependent tools less effective in prokaryotes, especially for 5mC with weak kinetic signals. These limitations underscore the need for approaches that are motif-flexible, sensitive to weak signals, and generalizable across diverse prokaryotic genomes and sequencing platforms.

To address these challenges, we propose KinMethyl, a learning-based framework that incorporates a signal modeling step to estimate baseline kinetic behavior from unmethylated DNA. By pretraining a regression model on whole-genome amplified (WGA) samples where methylation is absent, we capture the expected kinetic profiles for unmodified bases. These predicted values are then integrated with observed kinetic and sequence features in a downstream classifier, enhancing robustness under weak or noisy signal conditions. We employ a bidirectional gated recurrent unit (BiGRU)-based architecture and focal loss to handle label imbalance and improve detection accuracy.

We demonstrate that KinMethyl achieves strong performance in detecting 5mC in prokaryotic genomes across multiple motifs, species, and sequencing platforms, and extends successfully to other methylation types, including 6 mA and 4mC. Our results suggest that modeling expected kinetics is a powerful strategy for improving methylation detection in low-signal regimes, offering a practical solution for epigenomic analysis in diverse bacterial systems.

## 2 Methods

### 2.1 Problem formulation and overview

KinMethyl is designed for de novo methylation detection, where the underlying motif is unknown. This makes the method suitable for analyzing novel prokaryotic species or metagenomic data, in which methylation patterns have not been characterized in advance. We formulate the detection of 5-methylcytosine (5mC) from SMRT sequencing data as a binary classification problem. Given a genomic region centered on a candidate cytosine, along with polymerase kinetics signals (IPD and PW), the goal is to predict whether the site is methylated or unmethylated.

This task is particularly challenging in bacterial genomes due to weak kinetic signals, limited training data, and the diversity of methylation motifs. Direct classification using raw sequence and kinetic inputs risks overfitting to motif-specific patterns and sequencing noise. To address these challenges, we adopt a two-stage framework:

1. Signal modeling stage: A regression model is trained on WGA DNA, which lacks methylation, to predict expected IPD and PW values for unmethylated sequences.
2. Classification stage: The classifier receives raw kinetics, predicted kinetics from the regression model, and DNA sequence features, and outputs a binary methylation label.

This design enables the model to distinguish subtle kinetic alterations indicative of methylation by referencing predicted unmethylated baselines.

### 2.2 Data collection and preprocessing

We curated publicly available SMRT sequencing datasets from multiple bacterial species, including *Escherichia coli* K12, *Klebsiella pneumoniae*, *Oceanobacillus crateris* K2, and *Rhodobacter blasticus*, along with WGA-treated human and bacterial samples (Table 1). Known methylation motifs were derived from published annotations and include 5mC motifs (e.g. C**C**WGG, G**C**NGC), 6 mA motifs (e.g. G**A**TC), and 4mC motifs (e.g. GTA**C**, GGAT**C**C).

**Table 1.** Datasets used in this study.

Organism	Treatment	Motif	Methylation type	Platform	Chemistry	Reference
*K. pneumoniae*	Natural	C**C**WGG, G**A**TC	5mC, 6 mA	RSII	P6-C4	(Fu *et al.* 2022)
*O. crateris*	Natural	G**C**NGC, GGAT**C**C, ATTA**A**T	5mC, 4mC, 6 mA	RSII	P6-C4	(Fomenkov *et al.* 2020)
*E. coli* K12	Natural	C**C**WGG, G**A**TC	5mC, 6 mA	RSII	P6-C4	(McIntyre *et al.* 2019)
Human HEK293	WGA	None	None	RSII	P6-C4	(Kong *et al.* 2022)
*C. difficile*	WGA	None	None	RSII	P6-C4	(Zhu *et al.* 2018)
*R. blasticus*	Natural	GTA**C**	4mC	RSII	P6-C4	(Anton *et al.* 2018)
Human HEK-WGA	WGA	None	None	Sequel II	Sequel 2.1 polymerase	(Kong *et al.* 2022)
Human HEK-WGA-M.SssI	M.SssI	**C**pG	5mC	Sequel II	Sequel 2.1 polymerase	(Kong *et al.* 2022)
*E. coli* K12 MG1655	Natural	C**C**WGG	5mC	Sequel II	Sequel 2.1 polymerase	(Kong *et al.* 2022)

The bold letter in each motif represents the methylated base. In “Treatment” column, “Natural” means native samples from each organism without extra treatment, “WGA” means whole genome amplification where all methylations are eliminated, and “M.SssI” means all CpG sites are methylated as 5mC by M.SssI methyltransferase. Only motifs used in our computational experiments are shown. Further details on the datasets are described in Table S1.

Sequencing chemistries and platforms varied across datasets. For data generated using the PacBio RSII platform with P6-C4 chemistry, raw bax.h5 files were converted to bam format using bax2bam (Pacific Biosciences, v0.0.9) and aligned to reference genomes using pbmm2 (Pacific Biosciences, v1.13.1). Kinetic features were extracted using a custom pipeline adapted from ccsmeth, extended to support non-CCS reads and base-wise signal extraction. For Sequel II CCS datasets, we used the native ccsmeth feature extraction tool.

Raw IPD and PW values were normalized and standardized across all bases in the same reads as described in Supplementary Note. For each candidate cytosine, multiple subread measurements were averaged to reduce stochastic variation.

Positive samples were defined as 21-bp windows centered on methylated cytosines. Negative samples were selected differently for training and testing: In training, we used the same motif instances from WGA data (methylation-free). In testing, we selected random genomic regions that did not overlap known methylation motifs. Class balance was maintained in test sets to avoid performance inflation due to skewed label distributions.

### 2.3 Unmethylated signal modeling

**Figure 1.**
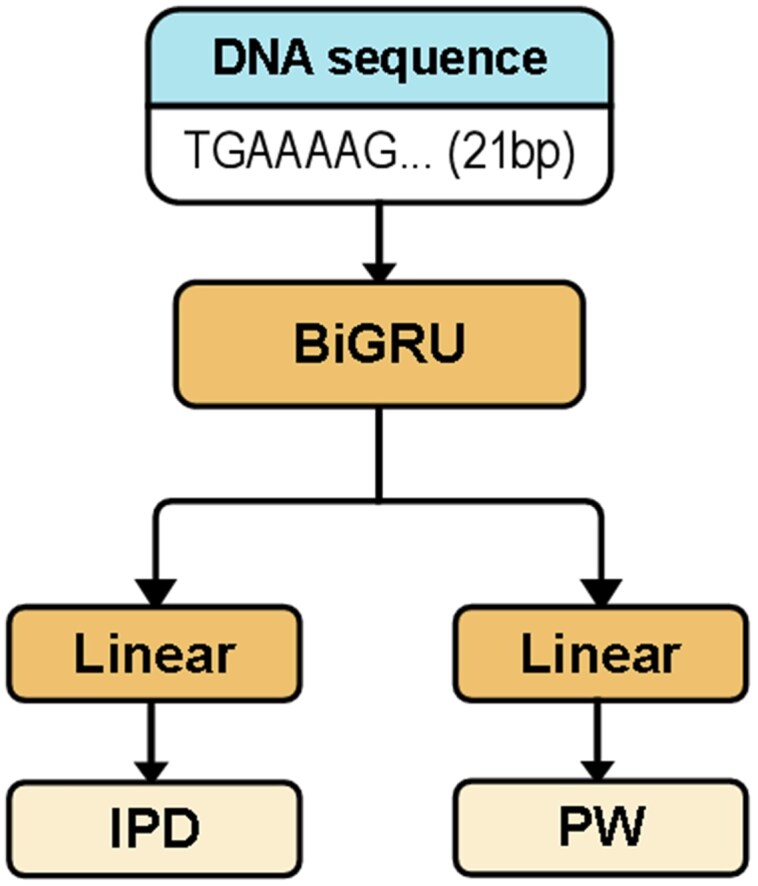
Network architecture of the regression model for the kinetic signals of unmethylated sequences.

To address the scarcity of positive samples and mitigate overfitting, we first trained a regression model to predict expected kinetic signals under the assumption of unmethylated DNA (Fig. 1). This model learns the relationship between local sequence context and polymerase kinetic signals, effectively capturing the baseline behavior of unmethylated cytosines.

The regression model was trained using WGA data, which lack methylations. Each input consists of a 21-base-pair sequence window centered on a cytosine. The model outputs predicted IPD and PW values for the central base. We used a BiGRU (Cho *et al.* 2014) encoder followed by two independent linear heads, one for IPD and the other for PW prediction.

The model was trained using the mean squared error (MSE) loss between predicted and observed kinetic signals. Once trained, this model serves as a frozen component during classification, supplying baseline (unmethylated) kinetic signals for each candidate site.

### 2.4 Methylation classification model

**Figure 2.**
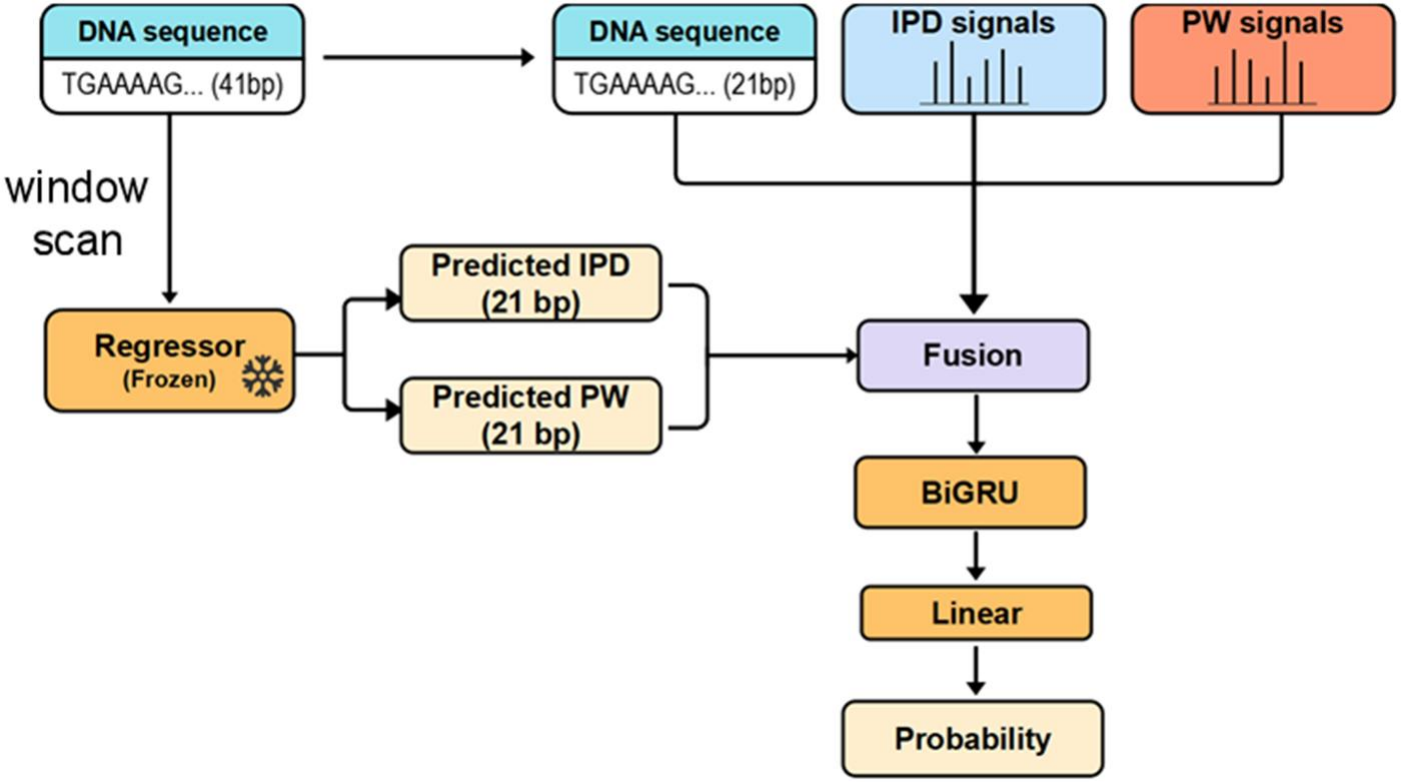
Network architecture of the methylation classification model.

The classification model integrates three types of features (Fig. 2):

1. Raw kinetics: IPD and PW values directly extracted from sequencing reads.
2. Predicted kinetics: Regression-estimated IPD and PW values representing unmethylated baselines.
3. DNA sequence: One-hot encoded representation of a 41-bp window centered on the candidate base.

Although the sequence window is 41 bp, only the central 21 bp are considered for classification. The full 41-bp sequence is processed by the pretrained regression model to generate predicted kinetic values for the center region.

We tested multiple feature integration strategies as described in the next section. In the main approach, raw and predicted kinetic signals and the sequence features are concatenated and passed into a BiGRU encoder. The output is fed into a fully connected layer to produce the final binary methylation prediction.

For CCS-based Sequel II data where predicted signals tend to be noisier, we adopted a multi-branch late fusion strategy. In this architecture, raw kinetics, predicted kinetics, and sequence features are encoded by separate BiGRU branches, and their outputs are merged at a higher level before classification. This design allows each modality to retain its own representation and reduces interference from noisy components.

### 2.5 Feature integration strategies

We systematically evaluated nine integration strategies as summarized in Table 2. These include the difference and the ratio between raw and predicted kinetic signals (IPD and PW). As ablation experiments, we also evaluated strategies with or without predicted kinetic signals and with or without sequence features. As shown in Results, the best-performing configuration (ID 6 in Table 2) included all three elements: difference, ratio, and sequence.

**Table 2.** Feature integration strategies.

ID	Kinetics difference	Kinetics ratio	Sequence
1	Y	N	N
2	N	Y	N
3	Y	Y	N
4	Y	N	Y
5	N	Y	Y
6	Y	Y	Y
7	Raw kinetics + Sequence		
8	Raw kinetics + Predicted kinetics		
9	Raw kinetics + Predicted kinetics + Sequence		

Kinetics difference is defined as raw kinetics-predicted kinetics while Kinetics ratio is defined as $\frac{\mathrm{raw}\;\mathrm{kinetics}}{\mathrm{predicted}\;\mathrm{kinetics}}$. In the strategy ID 8 and 9, the feature is directly concatenated without subtraction or division. In the strategy ID 7, predicted kinetic signals are not included, thus it can be seen as an ablation experiment not using unmethylated signal modeling. Y: used, N: not used.

### 2.6 Loss function and optimization

The regression model for unmethylated signal prediction was trained using MSE loss between predicted and observed kinetic signal values (IPD and PW) on unmethylated samples. Training was performed for 50 epochs using the Adam optimizer with a learning rate of 10^−3^ and a batch size of 64.

For the methylation classification model, we adopted two regularization strategies to improve robustness under class imbalance and label uncertainty:

1. Focal loss (Lin *et al.* 2018) with focusing parameter γ = 2 to prioritize difficult-to-classify examples:
  (1) $$\mathrm{FocalLoss}(p_{t})=-{\left(1-p_{t}\right)}^{\gamma }\;\log(p_{t}),$$
  where $p_{t}=p$ if the true label is 1 (methylated) and $p_{t}=1-p$ otherwise.
2. Label smoothing (smoothing factor = 0.1), which softens one-hot labels to prevent overconfident predictions.

Classification models were trained using Adam with the same learning rate of 10^−3^ with a learning rate decay (StepLR) of 0.5 every 10 epochs. All feature integration strategies were trained under identical settings to ensure comparability.

To improve decision stability, the classifier output scores were calibrated by threshold tuning. Instead of using a fixed cutoff (e.g. 0.5), we selected the threshold that maximized the F1 score on a separate validation set. This adjustment improved performance, especially in cross-organism evaluations where score distributions may vary.

### 2.7 Evaluation metrics

We used the Pearson’s correlation coefficient and the coefficient of determination *R*^2^ to evaluate the regression model for unmethylated kinetic signals (predicted vs. observed IPD/PW). To evaluate the methylation classification models, we used standard measures including accuracy (ACC), precision, recall and the area under the ROC curve (AUC).

We also employed integrated gradients (IG) (Sundararajan *et al.* 2017) to interpret the regression model by attributing predicted IPD/PW values to input nucleotide positions. This analysis revealed the relative contribution of surrounding bases to kinetic signal prediction.

### 2.8 Baseline methods

To benchmark our model, we compared against two established tools: kineticstools and ccsmeth.

In kineticstools, for RSII datasets, we used the analysis protocol RS_Modification_and_Motif_Analysis in SMRTanalysis v2.3.0 and extracted “IPDRatio” values from the output file modifications.csv. For Sequel II datasets, we used SMRTlink v7.0 to align reads with blasr, and to extract kinetic signal values with ipdSummary using the “SP3-C3” model.

Since ccsmeth is designed for CCS/HiFi reads, we only used for Sequel II datasets. We used the model “model_ccsmeth_5mCpG_call_mods_attbigru2s_b21.v3.ckpt” from ccsmeth v0.5.0 with the default modification calling pipeline.

Our method and these baselines methods were evaluated on the same datasets and with the identical definitions of positive/negative sites for fair comparison.

## 3 Results

### 3.1 Unmethylated signal modeling enables accurate reconstruction of kinetics from sequence

We first trained a regression model to predict kinetic signals (IPD and PW) of unmethylated DNA from sequence information. The model was trained on WGA data where all methylations are absent.

**Figure 3.**
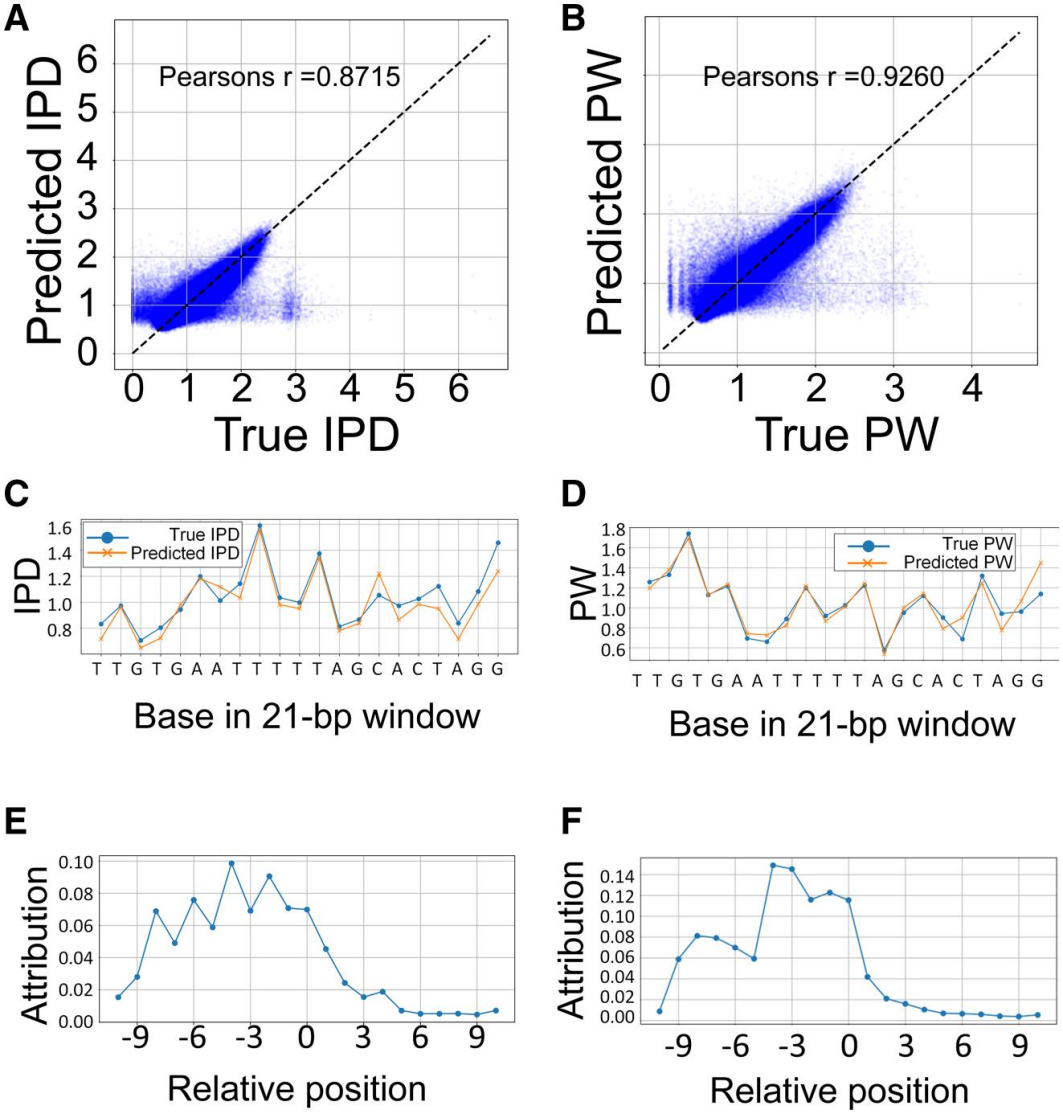
Performance of the regression model for the kinetic signals of unmethylated sequences. (A, B) Comparison between true and predicted kinetic signals (A: IPD, B: PW). (C, D) True and predicted kinetic signals along with an example 21-bp sequence TTGTGAATTTTTAGCACTAGG (C: IPD, D: PW). (E, F): IG attribution scores along with the 21-bp window averaged over different sequences (E: IPD, F: PW). Higher scores indicate the corresponding sequence position more contributed to kinetic signal prediction.

Across bacterial datasets, the predicted IPD and PW values showed high concordance with observed signals from unmethylated samples (Fig. 3). Pearson’s correlation coefficients exceeded 0.87 for IPD and 0.92 for PW. The model effectively captured sequence-dependent patterns, with slight deviations at extreme values likely attributable to sequencing noise (Flusberg *et al.* 2010). Attribution analysis using IG confirmed that the model relied on a symmetric window of flanking bases, consistent with prior findings (Feng *et al.* 2013). These results confirm that the regression model successfully learns kinetic signal patterns, providing a stable reference for downstream methylation classification.

### 3.2 Feature integration improves methylation classification

**Figure 4.**
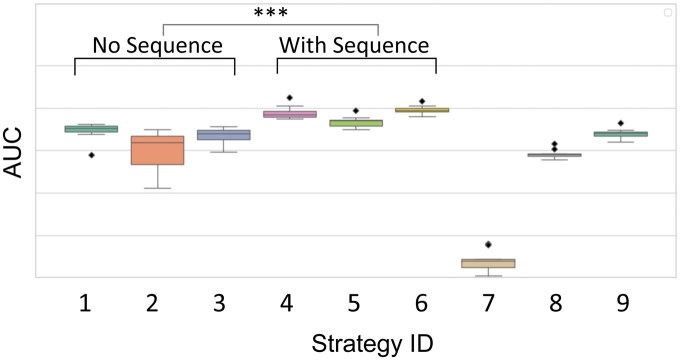
Comparison of classification performance (AUC) across different feature integration strategies. The strategy IDs correspond to those described in Table 2.

We evaluated the impact of various input features on classification performance using *K. pneumoniae* as the training species and *O. crateris* as the test species (Table 3 and Fig. 4). To assess the robustness of the results, we performed 10 rounds of bootstrap resampling to randomly partition the training data into training and validation sets, and repeated the training and evaluation process for each resampling. Nine different feature combinations (Table 2) were tested, including raw and predicted kinetic signals and sequence features.

**Table 3.** Comparison of classification performance (AUC, ACC, precision, recall, and AUPR) across different feature integration strategies.

Strategy ID	AUC	Acc	Precision	Recall	AUPR
**1**	0.8601	0.7275	0.6704	0.8992	0.8728
**2**	0.8341	0.6222	0.5760	0.9584	0.8402
**3**	0.8485	0.6863	0.6280	0.9222	0.8604
**4**	0.8978	**0.7867**	**0.7357**	0.9015	0.8999
**5**	0.8763	0.7472	0.6825	0.9310	0.8710
**6**	**0.9066**	0.7774	0.7162	**0.9294**	**0.9104**
**7**	0.5563	0.5456	0.5357	0.6798	0.5274
**8**	0.8053	0.6122	0.5677	0.9592	0.8020
**9**	0.8419	0.6299	0.5804	0.9724	0.8394

The strategy IDs correspond to those described in Table 2. Bold letters represent the best strategy for each evaluation measure. The averages of 10 rounds of bootstrap resampling are shown. The performance for each bootstrap is shown in Table S2.

The best AUC was achieved using the strategy ID 6 (Fig. 4), which combines raw-predicted kinetic signal differences, ratios, and sequence features. This configuration yielded an AUC of 0.9066, accuracy of 0.7774, and precision of 0.7162 (Table 3).

In addition, based on the pairwise comparisons of AUC values between strategies (Fig. S1, Table S3), several key trends emerged. Adding sequence information consistently improved performance (IDs 4, 5, 6 vs. IDs 1–3) (Mann-Whitney U test, *P *= 8.88 × 10^−10^). Both difference and ratio features enhanced discrimination independently, with their combination yielding the highest gains. Using only raw kinetic signals and sequence (ID 7) performed poorly (AUC = 0.5563), highlighting the importance of predicted unmethylated signals from the regression model.

These results demonstrate that kinetic signal modeling and multi-feature integration are essential for robust 5mC detection, particularly under weak signal conditions. In later sections, we report the results obtained by the strategy ID 6 as our method.

### 3.3 Our method achieves robust methylation detection in diverse bacteria and methylation motifs with cross-species generalizability

**Figure 5.**
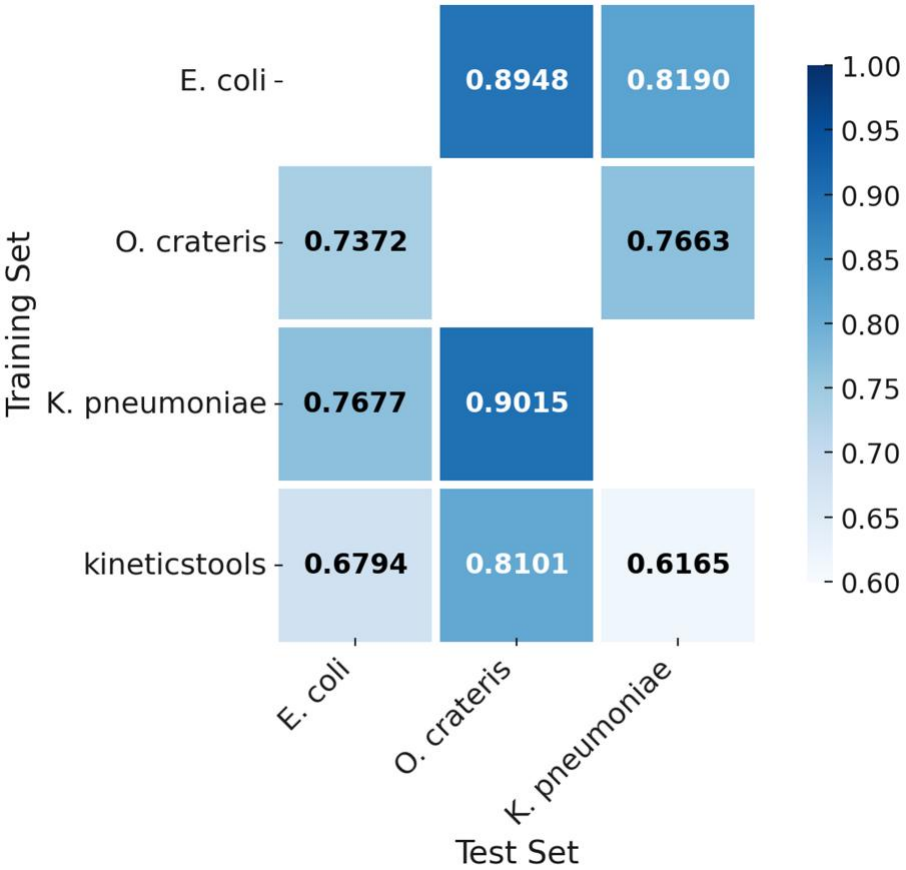
Methylation classification performance on 5mC with different training and test datasets. The performance of kineticstools is also shown as a baseline.

We assessed the robustness and generalizability of our method across bacterial species with diverse methylation motifs (Fig. 5, Table S4). Specifically, we conducted cross-species evaluations involving *E. coli* and *K. pneumoniae* (both with C**C**WGG motifs) and *O. crateris* (with G**C**NGC motifs). Models were trained on one species and directly applied to others without retraining or parameter adjustment.

Our method consistently achieved strong classification performance across species and motif types. For example, a model trained on *K. pneumoniae* achieved AUCs of 0.7677 on *E. coli*, showing a certain generalizability between species with a common motif sequence. More strikingly, our method showed the generalizability across different motif sequences. For example, even when trained on *O. crateris* (G**C**NGC motif), the model generalized well to C**C**WGG motifs in *E. coli* (AUC = 0.7372) and *K. pneumoniae* (AUC = 0.7663), despite motif and taxonomic divergence.

In contrast, kineticstools exhibited consistently lower performance in all species tested (Fig. S2). For example, its AUCs on the C**C**WGG datasets were only 0.6794 for *E. coli* and 0.6165 for *K. pneumoniae*. While it performed moderately better on *O. crateris* (AUC = 0.8101), it still fell short of our model, which achieved 0.9015 on the same test set.

### 3.4 Our framework extends to multiple methylation types and sequencing platforms

**Figure 6.**
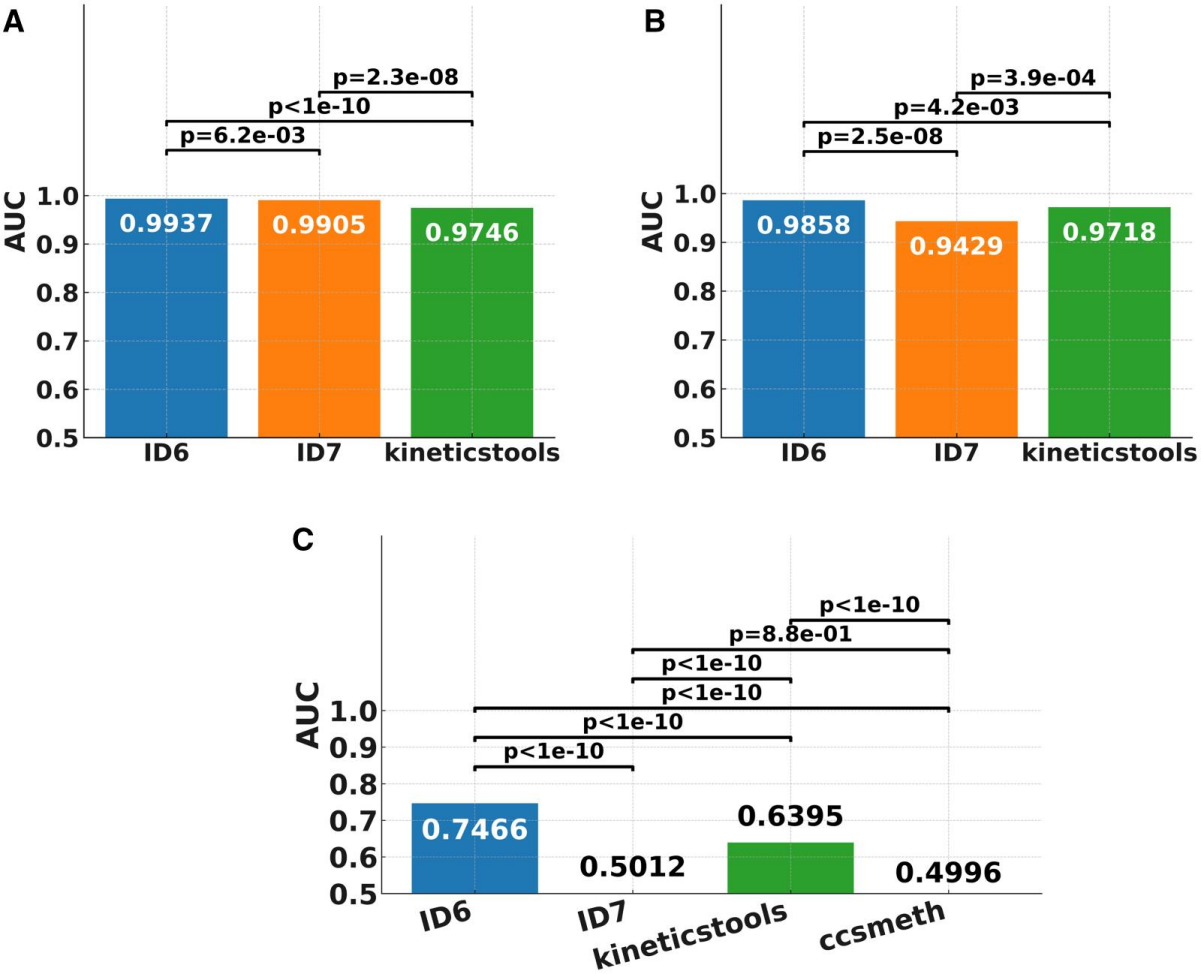
Methylation classification performance for different methylation types and sequencing platforms. A: Performance on 6 mA, *O. crateria* ATTA**A**T motif (RSII); B: Performance on 4mC, *O. crateria* GGAT**C**C motif (RSII); C: Performance on 5mC, *E. coli* C**C**WGG motif (Sequel). Our methods with the two feature integration strategies (IDs 6 and 7) were compared with kinetics tools and ccsmeth using DeLong’s test (Sun and Xu 2014). For 6 mA and 4mC, the performance of ccsmeth is not shown since it only supports 5mC detection.

To further evaluate the flexibility of our method, we applied the framework to additional methylation types (6 mA and 4mC) and newer sequencing platforms (Sequel II with CCS reads). These tests assessed whether the model architecture, originally designed for challenging 5mC detection, could also generalize to stronger kinetic signals and alternative polymerase chemistries (Fig. 6, Table S5).

In 6 mA detection tasks using RSII data from *O. crateris* ATTA**A**T motif, our method achieved near-perfect classification accuracy (Fig. 6A). With full feature integration (the strategy ID 6), AUC reached 0.9858, outperforming kineticstools (AUC = 0.9718) and the version without predicted kinetics (ID 7) maintained high performance (AUC = 0.9429). Similarly, in 4mC detection on *O. crateris* (GGAT**C**C motif) (Fig. 6B), our method achieved AUC = 0.9937 with full features, versus 0.9905 for the reduced-feature model and 0.9746 for kineticstools.

These results confirm that our architecture generalizes well to modifications with stronger kinetic signatures, even when predicted unmethylated signals provide limited additional benefit.

In contrast, 5mC detection from Sequel II with CCS reads presented a more challenging scenario due to lower signal-to-noise ratios and decreased regression accuracy (IPD *R*^2^ = 0.431, PW *R*^2^ = 0.758). Nevertheless, for the classification performance (Fig. 6C), integrating predicted signals using a late fusion strategy (ID 6) improved AUC from 0.5012 (raw kinetics and sequence only) to 0.7466, and outperformed kineticstools (AUC = 0.6395) and ccsmeth (AUC = 0.4996), highlighting the advantage of our architecture even in low-signal conditions. The limited generalizability observed for ID7 and ccsmeth may be due to their lack of predicted unmethylated signal integration, which is critical for capturing kinetic patterns. Notably, our model makes no assumptions about strand symmetry or motif palindromicity, unlike ccsmeth, which is optimized for symmetric CpG contexts. This makes our method more broadly applicable to prokaryotic methylation motifs where strand-specific kinetics are common.

Taken together, these results demonstrate that our method is robust across methylation types (5mC, 6 mA, 4mC), sequencing chemistries (RSII, Sequel II), and signal quality regimes. The ability to adapt to both strong and weak signals, while consistently outperforming existing tools, underscores the versatility and practical utility of our framework.

## 4 Discussion

### 4.1 Model design and feature integration

In this study, we presented a deep learning framework for methylation detection from SMRT sequencing that combines kinetic signal modeling with feature-integrated classification. The key innovation lies in modeling the expected kinetic signal of unmethylated DNA using a regression network pretrained on WGA data. This predicted baseline is then incorporated into a classifier that integrates raw kinetics, sequence context, and derived features such as kinetic signal differences and ratios.

Systematic ablation studies demonstrated that incorporating predicted kinetic signal alongside sequence and raw signals substantially improved classification accuracy, especially for 5mC, which is characterized by weak and context-dependent kinetic signals. Among the tested feature integration strategies, the combination of kinetic difference, kinetic ratio, and sequence (ID 6) consistently outperformed raw concatenation and other alternatives. These findings underscore the importance of thoughtful feature design and suggest that further architectural improvements (e.g. attention mechanisms or cross-modal transformers) could yield additional gains.

### 4.2 Versatility across species, modifications, and platforms

Our framework generalized effectively across species and methylation motifs. Cross-species experiments revealed that models trained on one bacterial genome often performed well on others, even across differing motifs such as C**C**WGG and G**C**NGC. This cross-motif transferability indicates that the model captures generalized kinetic patterns rather than overfitting to specific motifs or sequence contexts.

Furthermore, our method successfully extended to other methylation types. For 6 mA and 4mC, the model achieved near-perfect accuracy, confirming its applicability to modifications with stronger kinetic signals. In Sequel II CCS datasets, where regression quality degrades due to weaker kinetic signals, our model remained competitive by applying a late fusion strategy to minimize noise propagation. Notably, our framework outperformed both kineticstools and ccsmeth in all tested scenarios, including in the challenging context of strand-asymmetric prokaryotic methylation motifs.

These results emphasize the broad applicability of our method across taxa, motifs, and sequencing chemistries, making it a strong candidate for general-purpose methylation analysis in prokaryotic genomes.

### 4.3 Limitations and future directions

Despite its strengths, our approach has several limitations. First, while our framework improves 5mC detection, its absolute accuracy remains lower than that observed for 6 mA or 4mC. This discrepancy may stem from differences in signal intensity, as well as from imperfections in negative sample construction. For example, WGA-derived negatives used during training are strictly unmethylated, whereas negative test samples from native genomes may contain unannotated or partial modifications within the surrounding context window.

Second, our evaluation used class-balanced test sets, which may not fully reflect real-world deployment conditions where unmethylated sites vastly outnumber methylated ones. The risk of elevated false positives under imbalanced conditions will require further mitigation strategies, such as adaptive thresholding or post-hoc calibration.

Another challenge is the platform dependence of polymerase kinetics. As demonstrated in our experiments on Sequel II CCS datasets, changes in sequencing chemistry can reduce regression performance. Addressing this may require retraining the regression module for each polymerase version or designing architectures robust to such variability.

Future directions include expanding the framework to more methylation types and organisms. This study focused on DNA methylation, namely 5mC, 6 mA, and 4mC. But our two-step modeling approach may also improve the detection of base modifications other than methylation, such as phosphorothioation (Li *et al.* 2019; Cao *et al.* 2014). Future improvements could be achieved by incorporating sequencing quality into the analysis pipeline. For instance, selectively using high-coverage or high-quality reads, as demonstrated in a previous study (Ni *et al.* 2023), may enhance detection accuracy. Further improvements could also include improving interpretability via attribution and attention mechanisms, and leveraging semi-supervised or domain adaptation techniques to reduce dependence on labeled training data. As base-level methylation validation improves, further refinements in ground truth labels may also help enhance accuracy.

## Supplementary Material

vbaf249_Supplementary_Data

## Data Availability

The data underlying this article are available in the article and in its online supplementary material. KinMethyl software is available in GitHub: https://github.com/ZhangBio/KinMethyl.
