# Supplementary material for: KinMethyl: robust methylation detection in prokaryotic SMRT sequencing via kinetic signal modeling and deep feature integration
Source: Bioinform Adv. 2025 Oct 9;5(1):vbaf249. doi: 10.1093/bioadv/vbaf249 (PMC12552093; doi:10.1093/bioadv/vbaf249)
Supplement: vbaf249_Supplementary_Data [file vbaf249_supplementary_data.pdf]

Task	Species/Dataset	Selected Motif	Used as	Sample Number
K. pneu→O. cra	K. pneumoniae	C**C**WGG	Train/Positive	19813
	WGA set	C**C**WGG	Train/Negative	2049
	O. crateris	G**C**NGC	Test/Positive	11350
	O. crateris	Non-G**C**NGC	Test/Negative	11350
E.coli→O. cra	E.coli K12	C**C**WGG	Train/Positive	11588
	WGA set	C**C**WGG	Train/Negative	2049
	O. crateris	G**C**NGC	Test/Positive	11350
	O. crateris	Non-G**C**NGC	Test/Negative	11350
O. cra→E.coli	O. crateris	G**C**NGC	Train/Positive	19813
	WGA set	G**C**NGC	Train/Negative	2049
	E.coli K12	C**C**WGG	Test/Positive	11484
	E.coli K12	Non-C**C**WGG	Test/Negative	11450
K. pneu→E.coli	K. pneumoniae	C**C**WGG	Train/Positive	19813
	WGA set	G**C**NGC	Train/Negative	3191
	E.coli K12	C**C**WGG	Test/Positive	11484
	E.coli K12	Non-C**C**WGG	Test/Negative	11450
O. cra→K. pneu	O. crateris	G**C**NGC	Train/Positive	19813
	WGA set	G**C**NGC	Train/Negative	3191
	K. pneumoniae	C**C**WGG	Test/Positive	19107
	K. pneumoniae	Non-C**C**WGG	Test/Negative	18838
E.coli→K. pneu	E.coli K12	C**C**WGG	Train/Positive	11588
	WGA set	G**C**NGC	Train/Negative	3191
	K. pneumoniae	C**C**WGG	Test/Positive	19107
	K. pneumoniae	Non-C**C**WGG	Test/Negative	18838
6mA	K. pneumoniae	G**C**NGC	Train/Positive	31054
	WGA set	G**C**NGC	Train/Negative	603
	O. crateris	ATTA**A**T	Test/Positive	459
	O. crateris	Non-ATTA**A**T	Test/Negative	459
4mC	R. blasticus	GTA**C**	Train/Positive	8050
	WGA set	GTA**C**	Train/Negative	1162
	O. crateris	GGAT**C**C	Test/Positive	459
	O. crateris	Non-GGAT**C**C	Test/Negative	459
5mC(Sequel)	Human HEK-WGA-M.SssI	**C**G	Train/Positive	1227591
	Human HEK-WGA	**C**G	Train/Negative	1807792
	E. coli K12 MG1655	C**C**WGG	Test/Positive	70322/3006*
	E. coli K12 MG1655	Non-C**C**WGG	Test/Negative	71783/3006*

Task	Dataset	Sample Number
RSII signal modeling	WGA Set	7942987
SequelII signal modeling	Human HEK-WGA	301590447

Tables S1. The data used in the experiments. For RS datasets, kinetic features were aggregated at the site level prior to model training and prediction. For Sequel datasets, classification was performed at the subread-site level, and the resulting predictions were then averaged to obtain site-level scores for evaluation. *: number of subread-site/site.

Strategy ID	bootstrap_id	AUC	Acc	Prec	Reca	AUPR
1	1	0.8736	0.7688	0.7216	0.8752	0.8825
	2	0.8568	0.7215	0.6636	0.8984	0.8725
	3	0.8595	0.7452	0.6910	0.8869	0.8674
	4	0.8421	0.7213	0.6681	0.8796	0.8570
	5	0.8637	0.7282	0.6700	0.8993	0.8788
	6	0.8734	0.7275	0.6649	0.9174	0.8854
	7	0.8550	0.7047	0.6434	0.9184	0.8678
	8	0.8672	0.7127	0.6497	0.9232	0.8789
	9	0.8606	0.7389	0.6840	0.8880	0.8732
	10	0.8495	0.7062	0.6475	0.9052	0.8641
2	1	0.8497	0.5606	0.5326	0.9903	0.8554
	2	0.8401	0.6900	0.6293	0.9246	0.8471
	3	0.8757	0.6795	0.6150	0.9600	0.8830
	4	0.8413	0.6532	0.5958	0.9525	0.8479
	5	0.8628	0.5179	0.5091	0.9984	0.8698
	6	0.7850	0.6052	0.5626	0.9449	0.7896
	7	0.8464	0.6451	0.5890	0.9599	0.8514
	8	0.8360	0.6629	0.6051	0.9375	0.8442
	9	0.8333	0.6189	0.5701	0.9667	0.8383
	10	0.7706	0.5884	0.5513	0.9490	0.7756
3	1	0.8473	0.6189	0.5701	0.9678	0.8585
	2	0.8348	0.6587	0.6025	0.9328	0.8493
	3	0.8910	0.7198	0.6507	0.9490	0.8995
	4	0.8497	0.7098	0.6507	0.9057	0.8626
	5	0.8622	0.7067	0.6443	0.9230	0.8742
	6	0.8667	0.6789	0.6163	0.9482	0.8775
	7	0.8639	0.7115	0.6486	0.9235	0.8750
	8	0.7847	0.6807	0.6370	0.8401	0.7968
	9	0.8445	0.6857	0.6263	0.9210	0.8584
	10	0.8398	0.6924	0.6339	0.9109	0.8524
4	1	0.8947	0.7683	0.7017	0.9335	0.8942
	2	0.9007	0.7959	0.7410	0.9097	0.9029
	3	0.9000	0.7655	0.6975	0.9377	0.9004
	4	0.9061	0.7851	0.7201	0.9328	0.9082
	5	0.8920	0.7859	0.7331	0.8992	0.8946
	6	0.9128	0.8018	0.7436	0.9213	0.9173
	7	0.8977	0.7963	0.7457	0.8992	0.8970
	8	0.9169	0.8057	0.7467	0.9253	0.9200
	9	0.8716	0.7885	0.8094	0.7546	0.8758
	10	0.8854	0.7737	0.7178	0.9019	0.8889
5	1	0.8830	0.7647	0.7007	0.9242	0.8779

	2	0.8698	0.7406	0.6739	0.9323	0.8636
	3	0.8649	0.7011	0.6331	0.9569	0.8592
	4	0.8984	0.7828	0.7189	0.9289	0.8960
	5	0.8738	0.7442	0.6773	0.9328	0.8686
	6	0.8643	0.7304	0.6633	0.9359	0.8577
	7	0.8811	0.7533	0.6865	0.9326	0.8764
	8	0.8861	0.7410	0.6695	0.9519	0.8812
	9	0.8698	0.7793	0.7352	0.8732	0.8637
	10	0.8718	0.7350	0.6663	0.9416	0.8652
6	1	0.9052	0.7925	0.7345	0.9162	0.9099
	2	0.8946	0.6630	0.5998	0.9793	0.8996
	3	0.9220	0.7925	0.7237	0.9464	0.9250
	4	0.9078	0.7832	0.7167	0.9366	0.9097
	5	0.8994	0.7710	0.7058	0.9293	0.9025
	6	0.9139	0.7978	0.7359	0.9291	0.9179
	7	0.9100	0.7850	0.7190	0.9356	0.9130
	8	0.9088	0.8025	0.7491	0.9097	0.9131
	9	0.8905	0.7861	0.7389	0.8848	0.8967
	10	0.9135	0.7999	0.7390	0.9273	0.9164
7	1	0.5402	0.5341	0.5291	0.6200	0.5060
	2	0.5648	0.5525	0.5395	0.7159	0.5343
	3	0.5519	0.5425	0.5343	0.6629	0.5270
	4	0.6003	0.5755	0.5558	0.7511	0.5766
	5	0.5349	0.5314	0.5261	0.6341	0.5039
	6	0.5731	0.5571	0.5421	0.7352	0.5453
	7	0.5481	0.5350	0.5323	0.5774	0.5120
	8	0.5194	0.5207	0.5161	0.6656	0.4990
	9	0.5529	0.5474	0.5337	0.7501	0.5231
	10	0.5770	0.5602	0.5481	0.6852	0.5467
8	1	0.7861	0.6124	0.5671	0.9495	0.7843
	2	0.7988	0.5513	0.5273	0.9891	0.7962
	3	0.8276	0.6070	0.5615	0.9767	0.8247
	4	0.7814	0.6751	0.6247	0.8773	0.7773
	5	0.7968	0.6117	0.5658	0.9603	0.7918
	6	0.8086	0.6033	0.5596	0.9702	0.8051
	7	0.7991	0.6059	0.5617	0.9640	0.7972
	8	0.8187	0.6117	0.5648	0.9737	0.8118
	9	0.8242	0.6354	0.5819	0.9624	0.8218
	10	0.8113	0.6080	0.5627	0.9688	0.8094
9	1	0.8189	0.5000	0.5000	1.0000	0.8170
	2	0.8230	0.6721	0.6119	0.9410	0.8191
	3	0.8575	0.6259	0.5734	0.9834	0.8574
	4	0.8437	0.6238	0.5721	0.9823	0.8388

	5	0.8363	0.5680	0.5368	0.9922	0.8349
	6	0.8566	0.7097	0.6429	0.9434	0.8533
	7	0.8591	0.6557	0.5950	0.9753	0.8564
	8	0.8409	0.6653	0.6038	0.9616	0.8383
	9	0.8329	0.6195	0.5697	0.9769	0.8313
	10	0.8498	0.6593	0.5985	0.9677	0.8470

Tables S2. Comparison of classification performance across different feature integration strategies. The detail version of Table 3, showing the performance for each bootstrap.

Strategy ID	1	2	3	4	5	6	7	8	9
**1**		2.45E-02	2.63E-01	5.40E-04	5.88E-03	3.87E-04	3.87E-04	3.87E-04	9.04E-03
**2**	2.45E-02		2.98E-01	4.66E-04	1.51E-03	3.87E-04	3.87E-04	2.45E-02	8.50E-01
**3**	2.63E-01	2.98E-01		5.40E-04	4.80E-03	4.66E-04	3.87E-04	3.17E-03	2.89E-01
**4**	5.40E-04	4.66E-04	5.40E-04		4.80E-03	1.82E-01	3.87E-04	3.87E-04	3.87E-04
**5**	5.88E-03	1.51E-03	4.80E-03	4.80E-03		5.40E-04	3.87E-04	3.87E-04	3.87E-04
**6**	3.87E-04	3.87E-04	4.66E-04	1.82E-01	5.40E-04		3.87E-04	3.87E-04	3.87E-04
**7**	3.87E-04	3.87E-04	3.87E-04	3.87E-04	3.87E-04	3.87E-04		3.87E-04	3.87E-04
**8**	3.87E-04	2.45E-02	3.17E-03	3.87E-04	3.87E-04	3.87E-04	3.87E-04		9.12E-04
**9**	9.04E-03	8.50E-01	2.89E-01	3.87E-04	3.87E-04	3.87E-04	3.87E-04	9.12E-04	

Tables S3. Pairwise *p*-value between different feature fusion strategies.

Task	AUC	ACC	Precision	Recall	AUPR
E.coli→O. cra	0.8948	0.6577	0.9703	0.3254	0.8983
E.coli→K. pneu	0.8190	0.7141	0.8240	0.5496	0.8013
O. cra→E.coli	0.7372	0.6701	0.6308	0.8227	0.7053
O. cra→K. pneu	0.7663	0.6629	0.6086	0.9264	0.7286
K. pneu→E.coli	0.7677	0.5579	0.5318	0.9774	0.7327
K. pneu→O. cra	0.9015	0.7705	0.7027	0.9375	0.9027

Tables S4. KinMethyl performance in all cross-species experiments. Task column shows the species used for training and test as train ⟶ test.

Task.	AUC	ACC	Precision	Recall	AUPR
6mA ID6	0.9937	0.9557	0.9271	0.9891	0.9927
6mA ID7	0.9905	0.7326	0.6528	0.9937	0.9912
4mC ID6	0.9858	0.8584	0.9940	0.7211	0.9865
4mC ID7	0.9429	0.6144	0.9646	0.2375	0.9340

Tables S5. KinMethyl performance in 6mA and 4mC experiments.

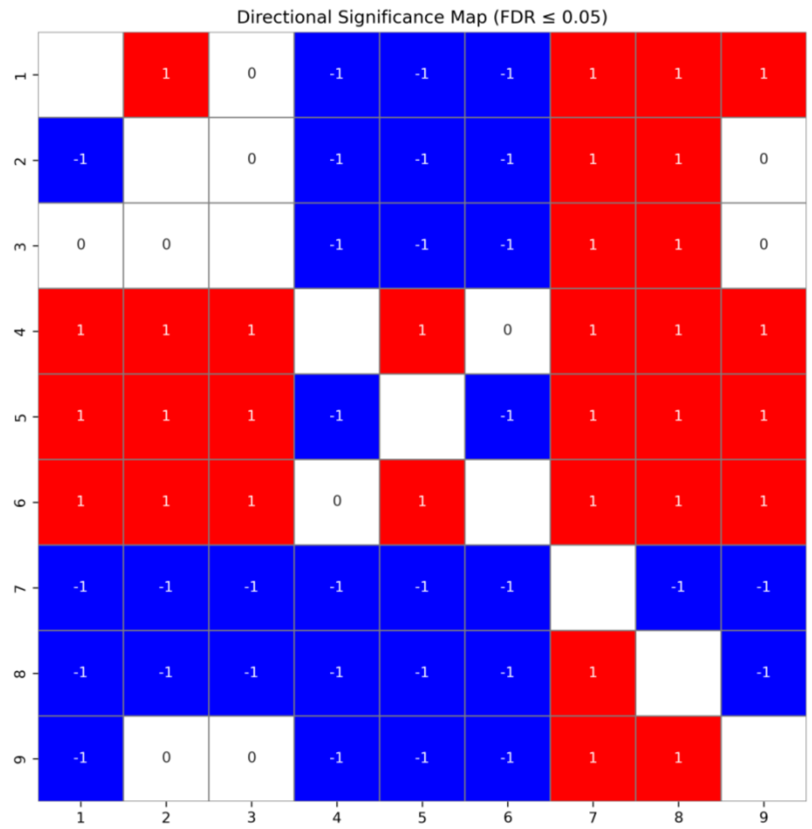

Figure S1. Pairwise statistical significance heatmap. Red cells indicate significantly better performance (FDR < 0.05) for the strategy listed in rows compared to that in the columns. Blue cells indicate significantly worse performance. White cells indicate no significant difference.

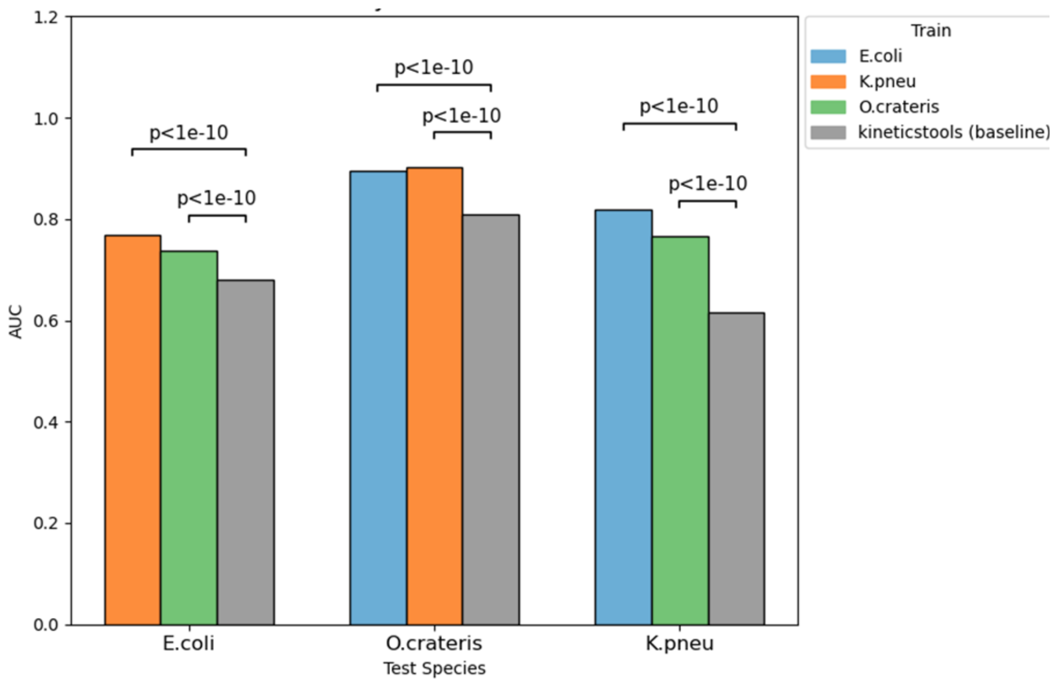

Figure S2. Comparison of kineticstools with KinMethyl with different combination of training and test species. The *p*-value is calculated using DeLong's test.

Supplementary Note

1 The normalization method of IPD and PW

We adopted 2 different normalization method when extracting IPD and PW from original sequencing data.

(1) Z-score normalization (used in ccsmeth and Sequel II experiments):

$$x' = \frac{x-\mu}{\sigma}$$

where x is the original IPD or PW value, μ is the mean, and σ is the standard deviation of the sample. x' is the normalized value (possibly negative).

(2) kineticstools-style normalization (used in kineticstools and our P6C4 experiments):

$$x' = \frac{min(x,\tau)}{\bar{x}}$$

where x is the original value, τ is the upper cap threshold (4·median(x) or a given percentile), $\bar{x}$ is the mean value after capping, and x' is the normalized value.

2 Statistical Comparison and p-value Calculation

To rigorously evaluate the significance of performance differences between models and integration strategies, we employed two statistical approaches:

(1) Feature Fusion: Mann–Whitney U test

For the comparison of feature integration strategies, we compared AUC distributions using a non-parametric approach. Specifically, for each strategy, we performed multiple training runs with different random seeds (i.e., bootstrap sampling). Pairwise statistical significance between the best-performing strategy and each alternative was assessed using the Mann–Whitney U test (Wilcoxon rank-sum test) based on the bootstrap AUC values. The resulting p-values were then adjusted for multiple comparisons using the Benjamini–Hochberg procedure to control the false discovery rate (FDR).

(2) Cross-Species and extending: DeLong’s Test

For direct comparison of model performance across species (cross-species experiments) and for extended tasks (such as other methylation types or sequencing platforms), we adopted the DeLong’s test, which is a widely used method for comparing the AUCs of correlated ROC curves. The DeLong test provides a p-value reflecting whether the difference in AUC between two models is statistically significant, taking into account the paired nature of predictions on the same test set.

3 Computational Efficiency

Model training and inference were performed using an NVIDIA H200 GPU. For our most computational-heavy experiment: 5mC Sequel Training on approximately 2.4 million samples for 20 epochs (batch size = 1024) required about 12000 seconds (~3.4 hours). Inference on a separate test set containing ~140,000 samples took

approximately 20 seconds.
